# The Occurrence of Bioactive Micromonosporae in Aquatic Habitats of the Sunshine Coast in Australia

**DOI:** 10.3390/md20080012

**Published:** 2008-06-05

**Authors:** Glen P. Eccleston, Peter R. Brooks, D. Ipek Kurtböke

**Affiliations:** Faculty of Science, Health and Education, University of the Sunshine Coast, Maroochydore DC, Qld 4558, Australia

**Keywords:** Actinomycetes, Micromonosporae, Bioactive compounds, Gentamicin, Aquatic habitats

## Abstract

Screening strategies based on the ecological knowledge of antibiotic producing microorganisms and their roles in the natural environment are being increasingly employed in the search for novel antibiotic agents. Micromonosporae are common inhabitants of aquatic habitats and have proved to be a continuing source of novel bioactive compounds including antibacterial and antitumor agents. The ecological distribution and frequency of bioactive micromonosporae in Sunshine Coast region aquatic habitats were studied through a range of selective isolation procedures designed to negatively select against the isolation of unwanted microbial taxa commonly associated with marine environments. It was revealed that bioactive compound producing species of micromonosporae were present in the aquatic habitats of the Sunshine Coast region in Australia.

## Introduction

The search for novel therapeutic agents for use in the pharmaceutical industry is driven by the need to combat the increase in the incidence of infection due to antibiotic resistant pathogens coupled with the search for novel antitumor and antiviral compounds [3, 20]. Natural products have been the major source of numerous therapeutic agents [16, 25] producing more than a half of the drugs in use today in many therapeutic categories [7].

Biotechnology companies have been engaged in natural product discovery using “ecological” or “bio-rational” approaches to detect specialized genes and metabolites that enable the survival of producing organisms in diverse and extreme environments [18, 46, 52]. For example, genome scanning of the deep-sea actinomycete ‘*Verrucosispora maris*” has produced an estimate of >20 biosynthetic gene clusters in this organism [13]. Highly selective isolation methods are also being used as part of objective but less conservative approaches undertaken towards the detection of bioactive compound producing taxa [26, 38, 48, 55, 67] given that successful discovery of novel bioactive compounds or chemical structures rests significantly upon the development of objective strategies for the isolation and characterization of such novel and rare microorganisms.

Within marine environments, taxonomically diverse bacterial groups exhibit unique physiological and structural characteristics that enable them to survive in extremes of pressure, salinity and temperature, with the potential production of novel secondary metabolites not observed in terrestrial microorganisms [13, 18, 49]. Much interest on the screening of marine and aquatic microorganisms is focused on screening sediment derived microorganisms [9, 14, 21, 32, 34, 47], and also on those that form highly specific symbiotic associations with marine plants and animals in response to the scarcity of nutrients in aquatic and marine environments, and thus produce compounds for defense and competition [36, 70].

Since the discovery of the antibiotic streptomycin in 1943 [57], actinomycetes have generally been the target of pharmaceutical research due to their ability to produce a diverse array of bioactive compounds [16, 28]. In particular, those actinomycetes of the family *Micromonosporaceae*, have been a rich source of bioactive molecules and the most prolific producers of anti-infective agents [42] behind the members of the genus *Streptomyces* from the production of antibiotic complex gentamicin in 1963 onwards [59] with a continuous stream of bioactive compounds including antibacterial and anti-tumor agents [30, 52]. Micromonosporae isolated from marine environments reportedly possess the highest rate of anti-tumor activity of the marine actinomycetes [70] including the recently detected cyctotoxic salinosporamides from *Salinispora* [61] and rifamycin from *Micromonospora* species [32] isolated from sediments.

Micromonosporae are frequent inhabitants of aquatic habitats worldwide where it is claimed they participate in the decomposition of cellulose, chitin and lignin [14, 24]. They have been isolated from water samples from streams, rivers and lakes, from lake mud, river sediments, beach sands, littoral sediments and deep marine sediments [14, 23, 33]. Micromonosporae have been found to be the dominant actinomycetes group in a range of aquatic environments, particularly in the deeper mud layers as well as in deep sea sediments [46, 51].

In Australia, significant efforts are underway to document the microbial diversity with industrial importance [4, 5, 11, 36, 37, 39]. The study of microbial flora associated with aquatic habitats and the screening of these organisms for valuable genetic material is receiving growing attention in the search for structurally diverse bioactive metabolites. Southeast Queensland offers a diverse array of aquatic habitats from saltwater estuaries and mangrove habitats to freshwater creeks and lakes for the study of occurrence and distribution of antibiotic-producing actinomycetes. In particular, the previously unscreened sub-tropical Sunshine Coast region can thus be seen as a key environment with a diverse range of biological resources to initiate ecological research into the habitats of those organisms that produce bioactive substances [40].

Rusnak *et al*., [52] have reported the isolation of a mixture of polycyclic aromatic compounds with activity against Gram-positive bacteria from a gentamicin producing species of *Micromonospora* stressing once more the fascinating biosynthetic potential of the taxon members in the production of diverse compounds. In line with this discovery the present study has investigated the occurrence of bioactive compound producing micromonoporae with particular emphasis on the gentamicin production from aquatic samples and sediments within Sunshine Coast habitats.

## Results

### Isolation and identification of Micromonosporae

**Figure 1:**
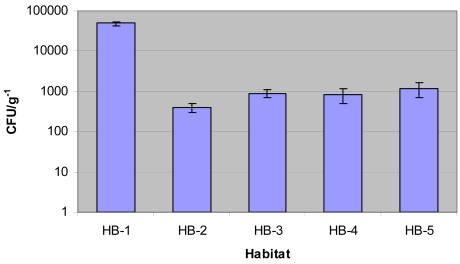
Average abundance of gentamicin-resistant actinomycete isolates (cfu) per gram of dried sediment sample.

Actinomycetes resistant to gentamicin were isolated from 20 of the 23 sediment samples collected at Sunshine Coast aquatic habitats on isolation media containing 1 μg/ml of gentamicin (Fig 1). Those sediments associated with habitat type HB-1, numerically displayed the largest populations of gentamicin-resistant actinomycetes, with a mean occurrence of 4.9 x 10^4^.g^−1^ of dried sediment. In HB-2, HB-3, HB-4 and HB-5, gentamicin-resistant actinomycetes were isolated on average between 4.0 x 10^2^ and 1.2 x 10^3^.g^−1^ of dried sediment.

**Figure 2:**
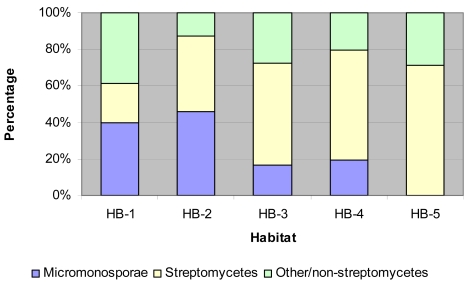
Composition of sediment derived actinomycetes associated with Sunshine Coast aquatic habitats. Micromonosporae Streptomycetes Other/non-streptomycetes

Micromonosporae were the predominant group of actinomycetes associated with HB-1 and HB-2, constituting 39.8% and 45.8% of the total gentamicin-resistant actinomycetes populations respectively. In HB-3 16.7% and HB-4 19.5% gentamicin-resistant micromonosporae isolates were detected. No micromonosporae isolates were recovered from the HB-5 (Fig 2).

Actinomycetes resistant to gentamicin were also isolated from six of nine lily-leaf specimens collected in Sunshine Coast aquatic habitats, predominantly on those lily-leaf samples in which decomposition was evident. Overall, 191 actinomycetes were isolated from lily-leaf specimens on isolation media containing 1 μg/ml gentamicin. Micromonosporae isolates, detected on all four of the decomposing lily-leaf samples, were found with a mean occurrence of 0.3 x 10^2^.g^−1^ of decomposing leaf sample, accounting for 9.8% of isolates, with the remaining 25.6% of isolates made up of actinomycetes identified as other, which belonged to neither micromonosporae nor streptomycetes.

**Figure 3:**
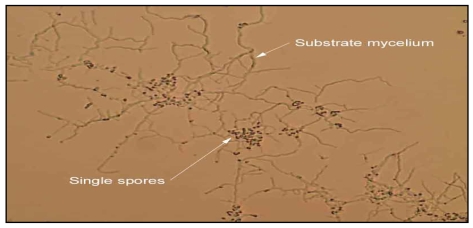
Light microscopic view of *Micromonospora* isolate USC-714, indicating single spore structures on a stable substrate mycelium typical of this genus.

In total, 75 actinomycetes from Sunshine Coast sediment and lily-leaf samples were identified as micromonosporae following their preliminary characterization (Fig 3). Forty-eight of these isolates were recovered from sediment samples on isolation medium containing 1 μg/ml of gentamicin, with a further 10 from lily leaf samples on this isolation medium. From those remaining isolates, five were isolated through preliminary sampling of sediments on isolation medium containing 5 μg/ml of gentamicin, and 12 without the addition of gentamicin to the isolation medium.

**Figure 4:**
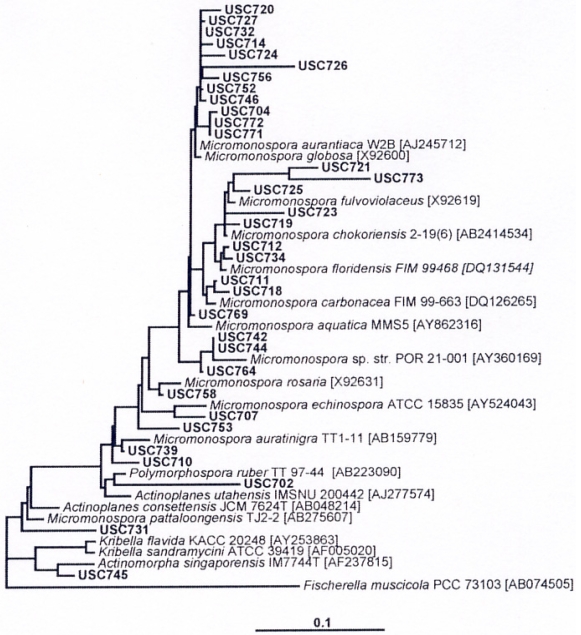
Phylogenetic tree of 16S rRNA gene sequences obtained from micromonosporae isolates compared against sequences obtained from public databases. The scale bar represents 10% sequence divergence. *Fischerella muscicola* was used as an out-group (GenBank accession numbers of reference sequences are included in brackets).

Further molecular sequencing indicated that the isolates belonged to the family Micromonosporaceae (Fig 4).

### Testing for antimicrobial activity

The antimicrobial activity of the isolates is shown in Table 3. All six antibiotic-producing *Micromonospora* control strains were shown to inhibit the growth of the Gram-positive test organism used in this study. The gentamicin-producing *M. echinospora* type strain (DSM 43816) was the only control strain shown to produce a broad spectrum of activity, capable of inhibiting the growth of both the Gram-positive and Gram-negative test organisms.

Only 12 of the Sunshine Coast isolates were capable of producing bioactive metabolites inhibiting the growth of either test organism (Table 4). Six of these antimicrobial compound producing isolates were recovered from the medium to which gentamicin had been added, with the remaining six isolates recovered during preliminary sampling from the medium without the gentamicin.

Only three isolates (USC-710, USC-731 and USC-758) displayed a broad spectrum of activity, inhibiting the growth of both the Gram-positive and Gram-negative test organisms, while only two isolates (USC-710 and USC-758) were also found to inhibit the growth of the VRE strain (Table 4). USC-710 and USC-731 were isolated from HB-1 and the USC-758 was isolated from HB-4.

### Chemical characterization of the bioactive compounds produced by the isolates

Antibacterial activity was persistent within the primary and secondary crude extracts deriving from the isolates USC-710, USC-731, USC-758 and the gentamicin-producing type strain *M. echinospora* (DSM 43816). The observed inhibition was found to be greatest in the primary extracts, compared to those produced from the secondary extracts.

**Figure 5:**
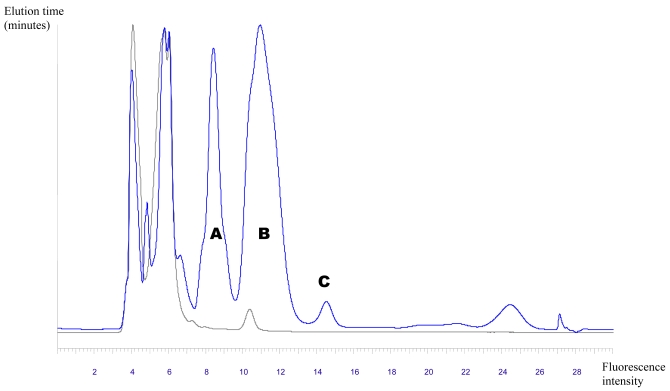
HPLC plot of derivatized gentamicin sulfate standard indicating gentamicin fractions (A, B and C) eluting at 8.4, 10.9 and 14.5 minutes: (a) absorption: 230 nm; (b) fluorescence: 455 nm.

Reversed-phase HPLC analyses of derivatized gentamicin-sulfate and the derivatizing reagent (OPA/N-acetyl-L-cysteine) alone, revealed fluorescent isoindole derivatives of gentamicin with OPA/N-acetyl-L-cysteine, separated into three fractions (A, B and C with retention times of 8.4, 10.9 and 14.5 minutes, respectively) that absorbed radiation at 230 nm and fluoresced at 455 nm (Figure 5). Absorbance at 230 nm and fluorescence at 455 nm is characteristic of isoindole derivatives, proving that the parent compound contained primary amine functions.

**Figure 6:**
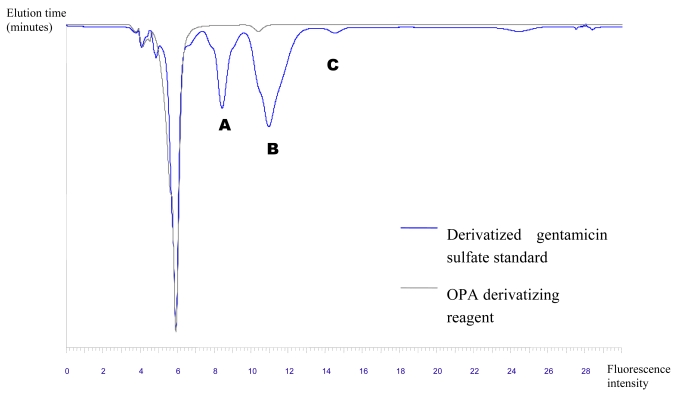
HPLC plot of DSM 43816 crude extract indicating peak eluting at 11.2 minutes: (a) absorption: 230 nm; (b) fluorescence: 455 nm.

When the HPLC traces of the derivatized crude and the non-derivatized crude extracts from DSM 43816 were compared with the gentamicin-sulfate standard, no corresponding absorbing and fluorescing peak was found within the non-derivatized sample. The derivatize extract of DSM 43816 displayed a new fluorescing peak at 11.2 minutes. This result indicated that gentamicin-like compounds were present in the crude extract obtained from the type strain (Figure 6). Whereas the HPLC analyses of the derivatized crude extracts from isolates USC-710, USC-731 and USC-758, revealed that no corresponding absorbing or fluorescing peaks were present in the region of the gentamicin standard. This indicates that none of the Sunshine Coast isolates were found to produce detectable levels of gentamicin or related primary amine antibiotic structures following HPLC analyses. The antibacterial activity associated with the crude extracts of these isolates is therefore not due to the activity of gentamicin or gentamicin-related compounds.

## Discussion

Following the application of the highly selective isolation techniques including selecting for gentamicin-resistance, micromonosporae were found to be the dominant actinomycete genus within Sunshine Coast aquatic sediments. Micromonosporae isolates were recovered from sediment samples collected at both marine and freshwater habitats, including littoral sands, river banks, creek beds and lake mud with the vast majority of micromonosporae and other actinomycetes isolates being obtained from mangrove mud habitats. These findings support the observations made by previous research towards the presence of bioactive compound producing actinomycetes [2, 32] in mangrove habitats which are highly productive marine ecosystems due to the high input of organic materials in the form of detritus. Through the breakdown of detritus materials, mangrove habitats support an abundant and diverse variety of organisms [1]. Micromonosporae isolates were reported to decompose complex organic compounds, such as cellulose, lignin and chitin that are not readily decomposed by the majority of aerobic bacteria and thus tend to accumulate in sediments [14]. Accordingly, micromonosporae found to be predominantly associated with mangrove mud sediments in this study can be seen as important components of benthic microbial communities within mangrove habitats, active in the degradation of complex organic materials.

Isolation of actinomycetes from decomposing lily-leaf samples revealed the predominance of actinomycetes including micromonosporae. These findings might provide further evidence towards the contribution of actinomycetes to the degradation of detritus and agree with the observation made by Goodfellow and Williams [24] in that micromonosporae and other genera of actinomycetes play an ecologically important role in the decomposition of plant materials, including cellulose and lignin, in aquatic ecosystems. The isolation procedures undertaken from leaf specimens subject to decomposition yielded greater densities of actinomycetes than those specimens without signs of decomposition. Wohl and McArthur [69] also reported that micromonosporae were found to be common isolates from dead leaf-tissues.

In comparison to mangrove mud sediments, those remaining benthic communities associated with littoral sand sediments, freshwater creek and lake habitats, yielded relatively low populations of gentamicin-resistant actinomycetes. In the analyses of benthic actinomycetes in freshwater lake sediments of the Sunshine Coast, micromonosporae were detected at a maximum concentration of 2.0 x 10^3^.g^−1^ of dried sediment. These findings also support the results of other researchers who reported correlations between the numbers of actinomycetes and organic matter content of that aquatic habitat [10, 14, 68].

Actinomycetes, in particular micromonosporae isolates are reported to be common inhabitants of lake habitats, especially in lake sediments [14], with the distribution and numbers of actinomycetes in lake habitats often reflecting the productivity status of the lake [35]. The comparatively low numbers of micromonosporae in Sunshine Coast freshwater habitats and littoral sand sediments may therefore be related to the low organic nutrient levels typical of these environments, compared to high nutrient habitats, such as those of mangrove mud.

Gause *et al.* [19] demonstrated that antibiotic-producing actinomycetes possess resistance determinants to self-produced antibiotics. Other researchers have reported a correlation between self-produced antibiotic resistance and the antibiotic production [17, 29, 31]. Consequently, gentamicin resistance has been used in rapid screening strategies for the isolation of antibiotic producing micromonosporae [5, 8, 31]. Although in this study micromonosporae isolated from the agar plates incorporated with gentamicin were not found to have an increased ability of producing diffusible bioactive metabolites compared to those isolated from the agar plates without gentamicin, the most bioactive isolates USC-731 and USC-758 were still isolated from the plates with gentamicin 1 μg/ml and 5 μg/ml respectively.

Isolation of bioactive compound producing micromonosporae in the Sunshine Coast aquatic habitats might be deriving from ecological functions of the microorganisms [64] rather than their resistance to gentamicin. According to Williams [62], antibiotic production is often associated with sites of high nutrient content as in areas rich in decaying organic matter, with antibiotic production evolving in response to selective pressures created through increased competition. Long and Azam [43] found in their study on antagonistic interactions among marine pelagic bacteria that a greater ability to chemically inhibit their free-living counterparts was a characteristic of particle attached bacteria. They suggested that the production of inhibitory metabolites play an important ecological role in deterring free-living bacterial competitors, aiding particle attached bacteria during competition for resources and involvement in biogeochemical cycling. This might relate to increased biological activities among micromonosporae associated with detritus in aquatic environments as well as with sediments.

Findings indicate that if target directed and highly selective isolation techniques are used, bioactive micromonosporae and other actinomycetes could be detected in mangrove and organic rich sediments. Such efforts can then contribute towards the national efforts in establishing information on the distribution of bioactive actinomycetes in Australian habitats [39, 40].

## Experimental Section

### Sampling Sites

A total of 23 aquatic sediment samples and nine lily-leaf samples were collected from 26 aquatic habitats around the Sunshine Coast, Queensland for the selective isolation of actinomycetes. Sediment sampling was conducted into sterile Petri-plates from the upper 20 mm of surface sediment, at water depths ranging down to 200 mm. Sample types were grouped into one of the six classifications, specified according to observations of marine or freshwater environment and sediment composition (Table 1).

### Selective Isolation of Micromonosporae

Combinations of selective pressures were applied to the samples to deselect common bacterial taxa on isolation plates. Air-dried (ambient room temperature for 14 days), ground and sieved sediment samples [27] were subjected to dry heat treatment at 55°C for 30 minutes (modified from Rowbotham and Cross [51]). Following the heat treatment, the sediment samples (2 g) were further microwave irradiated at 80 watts for 30 seconds [12]. Irradiated sediment samples were subjected to a 10 fold dilution series and aliquots (0.5 ml) from selected dilutions spread plated in triplicates onto the surface of the isolation media.

For the isolation of micromonosporae from lily-leaf samples surface sterilized and chopped leaf portions were placed into sterile water (9.0 ml) and shaken in a Flask-Shaker SF-1 (Stuart Scientific, UK) for 20 minutes (modified from Shimizu *et al*. [54]). Leaf suspensions were further subjected to sonication (modified from Wohl and McArthur [69]) for 1 minute in a sonicated water bath (Soniclean 160HT, Soniclean Pty. Ltd.). Sonicated leaf samples were further wet heat treated at 55°C for 60 min in a water bath (modified from Rowbotham and Cross [51]). A 10 fold dilution series was prepared of the heat treated samples in sterile water and aliquots (0.5 ml) from selected dilutions were spread plated in triplicates onto the surface of the isolation media.

The medium used for the isolation and enumeration of the gentamicin-producing micromonosporae from sediment and lily-leaf samples was Gause Mineral agar #1 [12], supplemented with the antifungal agents cycloheximide (50 μg/ml) and nystatin (50 μg/ml) [63]. Media was also supplemented with gentamicin-sulphate (1 μg/ml) to select for actinomycetes with resistance to gentamicin [19]. As a control treatment, isolation plates without gentamicin-sulphate were also used to test whether the incorporation of the antibiotic gentamicin (5 μg/ml) would affect the growth of the targeted taxon.

The isolation plates were inoculated with selected dilutions and air dried in a biological safety cabinet for 20 min [56]. The inoculated plates were incubated at 28°C for 28 days [65], following which the colonies were enumerated on the basis of the morphological characteristics of the previously described actinomycete taxa [22].

### Identification of the isolates

Purified isolates onto oatmeal agar [66] were further tentatively identified according to their characteristic morphology of sporulating structures and spore arrangements under light microscope using the cover slip technique [15]. Purified isolates were stored in 20% glycerol suspension placed into 2 ml cryogenic vials (In vitro Technologies Pty. Ltd., Australia) and kept at -20°C [60].

Isolates were further characterized using molecular sequencing [58] methods. For molecular characterization the PCR products were sequenced at the Australian Genome Research Facility, Brisbane. Once the sequence information is received the ARB program [45] was used for sequence alignment and phylogenetic analyses as described previously [50]. Sequences that were closely related to isolate 16S rRNA gene sequences were identified in ARB and were used in the analysis. Phylogenetic trees were calculated using evolutionary distance (Jukes and Cantor model), maximum parsimony (ARB and DNAPARS) and maximum likelihood (ARB and FASTDNAML) analyses of the aligned near full-length sequences [44]. Tree topologies generated using the three methods were compared and a tree representative of the overall consensus tree was selected. The phylogenetic tree presented is based on the evolutionary distance analysis.

### Testing for antimicrobial activity

Seventy one selected micromonosporae isolates were tested for their antimicrobial activities. Fifty nine of these isolates were recovered from isolation medium incorporated with gentamicin and the remaining 12 were the isolates from the medium without gentamicin incorporation.

Antibiotic producing type strains obtained from the DSMZ, Germany were used as control organisms (Table 2). Laboratory strains of Gram-positive (*Staphylococcus aureus*, penicillin sensitive, (Strain code: NA-1) and Gram-negative *(Escherichia coli* (Strain code: Hb 101)) and a Vancomycin resistant *Entrococcus* strain (VRE, clinical isolate obtained from a local hospital) were used to test the antimicrobial activity of the micromonosporae isolates.

Agar plugs were removed with a 5 mm diameter corer from 14 day old growing cultures of the actinomycetes on oatmeal agar medium. The surface growth on agar plugs was removed with a sterile scalpel blade, to obtain only the diffused microbial metabolites in the agar plugs. The agar plugs were inverted and placed onto nutrient agar plates inoculated with the test organism. The plates were then incubated at 37°C for 24 hours. Following incubation, antimicrobial activity was indicated by the formation of an inhibition zone surrounding the agar plug, which may provide an indication of diffused antimicrobial metabolites produced by the growing actinomycetes culture. The absence of an inhibition zone indicated a negative result for the production of diffusible metabolite into the solid growth medium.

### Chemical characterization of the bioactive compounds

Gentamicin is a basic, polar, broad-spectrum, antibiotic complex belonging to the aminoglycoside family of antibiotics, which contains primary amine functions that can be separated and analyzed by a High Performance Liquid Chromatography (HPLC) when appropriately derivatized [53]. Bioactive compounds produced by the micromonosporae isolates as well as the diffused antimicrobial metabolites of the gentamicin-producing *M. echinospora* type strain (DSM 43816) were analyzed using a HPLC (Perkin Elmer Series 200) for the presence of gentamicin-like compounds.

Ten selected isolates with displayed antimicrobial activity during the preliminary tests and the gentamicin-producing *M. echinospora* type strain (DSM 43816) were grown on oatmeal agar. Inoculated plates were incubated at 28°C for 14 days, allowing for the diffusion of antimicrobial products into the agar medium by the microorganisms (adapted from Kurtböke *et al*., [41]). Following incubation, the agar plates of each isolate were blended to form an agar mash. The antimicrobial metabolites were extracted by suspending the agar mash in 500 ml of methanol (MeOH) with 1 ml of acetic acid and keeping the blend overnight in a fume cupboard. The MeOH solvent containing diffused bioactive compounds were then separated from the agar mash by vacuum filtration. Excess MeOH and acetic acid was concentrated on a BÜCHI Rotavapor (Model R-205) under reduced pressure. The remaining aqueous filtrate formed the primary crude extract. Remaining agar mash was again blended and re-suspended in 200 ml of MeOH overnight, with a secondary aqueous crude extract obtained following filtration and evaporation.

### Testing of crude extracts for retention of antimicrobial activity

Crude extracts were tested for the retention of antimicrobial activity following extraction from the agar mash. The aqueous extracts were dissolved onto antibiotic susceptibility test disks (OXOID, Australia) and later the test disk was dried in a laminar flow cabinet until the solvent evaporated. Dried test disks were placed along with a gentamicin test disk (OXOID Australia, CN 30) and dried agar mash segments (as controls if the compound were not to be extracted and retained in the agar mash) onto nutrient agar plates inoculated with the *S. aureus*, *E. coli* and VRE test strains. These plates were incubated at 37°C for 24 hours and inhibition zones were recorded.

### HPLC analysis of crude extracts

Detection of gentamicin and gentamicin-related fractions in the crude extracts was undertaken by OPA/N-acetyl-L-cysteine derivatization and HPLC analysis. OPA/N-acetyl-L-cysteine forms isoindole derivatives of gentamicin which are UV absorbing at 230 nm and fluorescent at 455 nm [56].

The derivatised gentamicin-like compounds were separated by reverse phase HPLC and analyzed simultaneously at 230 nm and 455 nm. When derivatized with an OPA-N-acetyl-L-cysteine reagent, the derivatisation reagent yield is detectable by a corresponding absorbing and displayed fluorescence through HPLC analysis. The eluted derivatives (if any) were compared against those obtained following HPLC analysis of gentamicin-sulphate standard (Sigma-Aldrich G4793) The derivatizing reagent was prepared by dissolving 100 mg *o*-phthaldehyde (OPA) and 160 mg N-acetyl-L-cysteine in 4.5 ml MeOH, with 1.0 ml of 0.5 M sodium borate (pH 9.5) added to the suspension. The OPA reagent was stored in the dark at −20°C for a period not exceeding one week. The primary amine functions were derivatized by filtering 40 μl of the crude extract into a 2 ml amber vial into which 60 μl of the OPA reagent samples were added. The suspension was placed at ambient temperature in the dark for four minutes to allow for the reaction to occur.

Gentamicin derivatives were detected using a Perkin Elmer HPLC system, series 200-pump and diode array detector, equipped with an Alltech Spherisorb ODS-2 reverse phase column (Cat. No. 8736; Serial no. 92100087; length 250 mm; I.D. 4.6 mm), with a mobile phase consisting of a filtered 1:1 0.02 M NaH_2_PO_4_/0.01 M Na_2_HPO_4_ buffer : MeOH. The mobile phase was filtered (0.45 μm nylon membrane) and degassed prior to use. After four minutes sample reaction time, 20 μl of the mixture was injected with a mobile phase flow of 0.6 ml/min and 40 minute run time. Eluting compounds were detected at 230 nm and 455 nm and the data analyzed using Turbochrom Navigator © software on a Dell OptiPlex GX110.

## Figures and Tables

**Table 1:** Sunshine Coast aquatic sampling locations and habitat types.

Sample Locations	Habitat types	Habitat Descriptive Image
Petrie Ck, McDonald Rd, Diddillibah (S1) Paynter Ck, Diddillibah Rd, Diddillibah (S2) Maroochy R, River Store Rd, Maroochy (S3)	HB-1 Mangrove mud sediments derived from the Maroochy River or tributaries	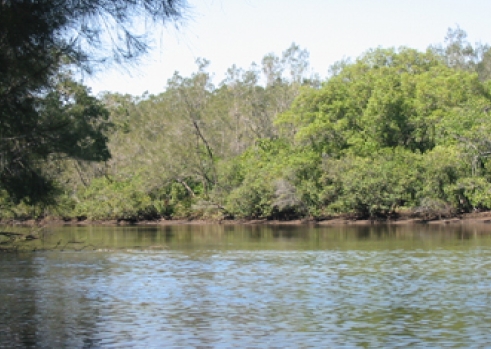
Maroochy R, Muller Park, Bli Bli (S4) Maroochy R, Oysterbank Rd, Bli Bli (S5) Maroochy R, Nojoor Rd, Mudjimba (S6) Eudlo Ck, Fishermans Rd, Maroochydore (S7)	HB-2 Sand sediments derived from the Maroochy River or tributaries	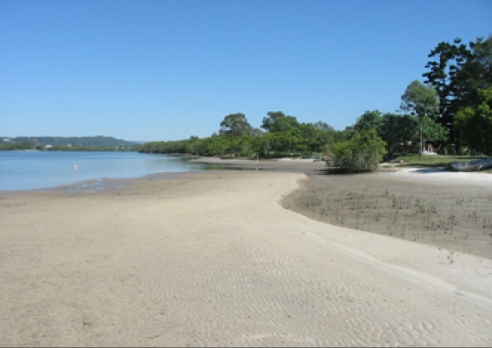
Stumers Ck, David Low Way, Coolum (S19) Coondibah Ck, Flinders St, Currumundi (S20) Currumundi Lake, Noel Burns Park, Currumundi (S21) Mooloolah R, Marra Ct, Mountain Creek (S22)	HB-3 Marine sand sediments not derived from the Maroochy River or tributaries	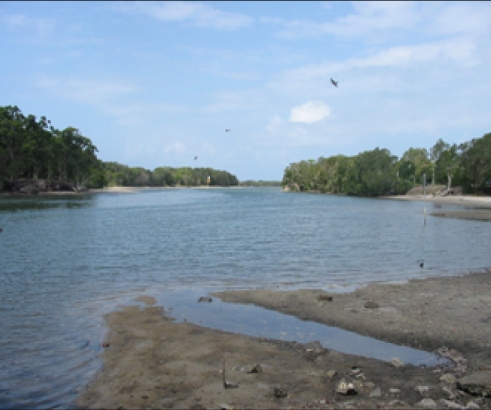
Lillyponds, Delicia Rd, Mapleton (S11) Tesch Park, Coral St, Maleny (S16) Ewen Maddock Dam, Glass House Mountains Rd, Landsborough (S17) Lake Macdonald, Noosa Shire Botanical Gardens, Pomona (S18) Wetland adjacent to David Low Way, Marcoola (S23)	HB-4 Fine, silty sediment from freshwater habitats	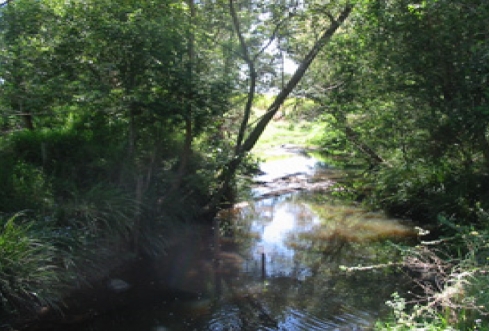
Obi Obi Creek Crossing no. 2, Obi Obi Rd, Kidaman Creek (S12) Little Yabba Ck, Little Yabba Creek Picnic Area, Conondale- Kenilworth Rd, Kenilworth (S13) Boolumba Ck, Dayuse Area - Area 2, Booloomba Creek Rd, Kenilworth (S14) Mary River, Maleny-Kenilworth Rd, Conondale (S15)	HB-5 Coarse grained sediment from freshwater habitats	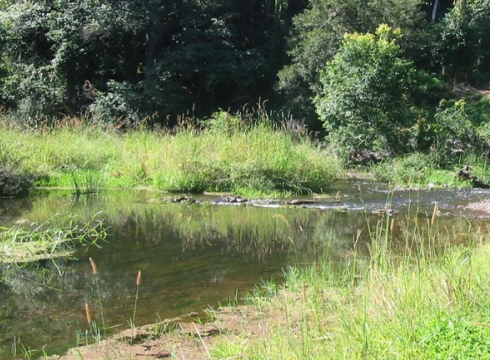
Wappa Dam, Wappa Dam Rd, Kiamba (L24) Wetland adjacent to David Low Way, Marcoola (L25) Hyatt Regency Lakes, David Low Way, Coolum (L26) Nelson Park Lakes, Alexandra Headland (L27) Lake Macdonald, Noosa Shire Botanical Gardens, Pomona (S18) Ewen Maddock Dam, Glass House Mountains Rd, Landsborough (S17) Wetland adjacent to David Low Way, Marcoola (S23)	HB-6 Lily-leaf samples from freshwater habitats	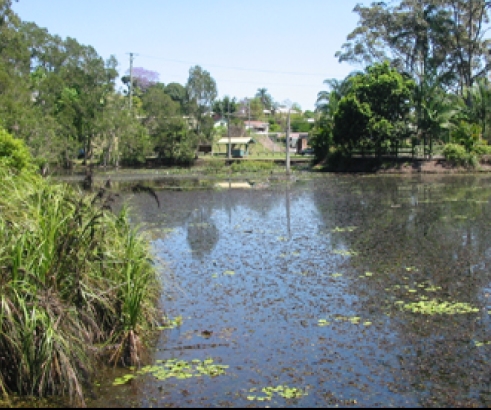

**Table 2:** *Micromonospora* type strains obtained from the DSMZ (*German Collection of Microorganisms and Cell Cultures*, Braunschweig, Germany, http://www.dsmz.de/)

DSM No.	Organism	Antibiotic produced	Isolated from
DSM 803	*M. rosaria*	Rosaramycin	Soil
DSM 43168	*M. carbonacea*	Everninomicins	Soil
DSM 43171	*M. halophytica*	Halomycin	Salt pool
DSM 43816	*M. echinospora*	Gentamicin	Soil
DSM 43817	*M. pallida*	Gentamicin	Soil
DSM 43818	*M. nigra*	Halomycin	Salt pool
DSM 43868	*M. olivasterospora*	Fortamicin B	Soil, paddy field

**Table 3:** Antibacterial activity of *Micromonospora* isolates from the Sunshine Coast aquatic habitats.

Original Isolation Media	Number of isolates screened for antibacterial activity	Antibacterial activity	Percent (%) of total
With gentamicin	59	6	10.2%
Without gentamicin	12	6	50.0%
Total	71	12	16.9%

**Table 4:** Activity spectra of the bioactive compounds extracted from the Sunshine Coast *Micromonospora* isolates.

Isolate no.	Antimicrobial activity		
	*S. aureus*	*E. coli*	VRE
USC-702 a	✓	X	X
USC-703 a	✓	X	X
USC-705 b	✓	X	X
USC-706 b	✓	X	X
USC-707 b	✓	X	X
USC-708 a	✓	X	X
USC-710 b	✓	✓	✓
USC-712 b	✓	X	X
USC-713 b	✓	X	X
USC-731 a	✓	✓	X
USC-757 a	✓	X	X
USC-758 a	✓	✓	✓

✓ Denotes inhibition of the growth of the test organism. X Denotes non-inhibition of the growth of the test organism.

a Denotes isolates obtained from media containing gentamicin.

b Denotes isolates recovered during preliminary sampling on isolation media without gentamicin.
